# Optical coherence tomography angiography to assess for retinal vascular changes in Neuro-Sjögren

**DOI:** 10.1177/25158414241294024

**Published:** 2024-10-31

**Authors:** Melanie Haar, Franz Felix Konen, Marten A. Gehlhaar, Irene Oluwatoba-Popoola, Emilia Donicova, Marija Wachsmann, Ahmed Lubbad, Katerina Hufendiek, Amelie Pielen, Bettina Hohberger, Christian Mardin, Stefan Gingele, Nils K. Prenzler, Diana Ernst, Torsten Witte, Carsten Framme, Thomas Skripuletz, Tabea Seeliger, Anna Bajor

**Affiliations:** Department of Ophthalmology, Hannover Medical School, Carl-Neuberg-Straße 1, Hannover 30625, Germany; Department of Neurology, Hannover Medical School, Hannover, Germany; Department of Ophthalmology, Hannover Medical School, Hannover, Germany; Department of Ophthalmology, Hannover Medical School, Hannover, Germany; Department of Ophthalmology, Hannover Medical School, Hannover, Germany; Department of Ophthalmology, Hannover Medical School, Hannover, Germany; Department of Ophthalmology, Hannover Medical School, Hannover, Germany; Department of Ophthalmology, Hannover Medical School, Hannover, Germany; Department of Ophthalmology, Hannover Medical School, Hannover, Germany; Department of Ophthalmology, Universitätsklinkum Erlangen, Erlangen, Germany; Department of Ophthalmology, Universitätsklinkum Erlangen, Erlangen, Germany; Department of Neurology, Hannover Medical School, Hannover, Germany; Department of Otolaryngology, Hannover Medical School, Hannover, Germany; Department of Rheumatology & Immunology, Hannover Medical School, Hannover, Germany; Department of Rheumatology & Immunology, Hannover Medical School, Hannover, Germany; Department of Ophthalmology, Hannover Medical School, Hannover, Germany; Department of Neurology, Hannover Medical School, Hannover, Germany; Department of Neurology, Hannover Medical School, Hannover, Germany; Department of Ophthalmology, Hannover Medical School, Hannover, Germany

**Keywords:** chronic inflammatory demyelinating polyneuropathy, neuro-Sjögren, optical coherence tomography angiography, retinal vasculature, Sjögren’s syndrome, vasculitis

## Abstract

**Background:**

Sjögren’s syndrome is an autoimmune disease characterized by sicca symptoms and various extraglandular manifestations including vasculitis. Neurological involvement occurs frequently (Neuro-Sjögren) and often mimics immune neuropathies such as chronic inflammatory demyelinating polyneuropathy (CIDP).

**Objectives:**

We aim to assess relevant differences in vessel density (VD) in Optical Coherence Tomography Angiography (OCTA) in those diseases to use it as an easily available diagnostic tool.

**Design:**

Prospective, monocentric pilot-study.

**Methods:**

OCTA (Heidelberg Engineering OCT SPECTRALIS) of the superficial vascular plexus, intermediate capillary plexus (ICP) and deep capillary plexus (DCP) of the retina was prospectively performed in Neuro-Sjögren, age-matched CIDP patients (*n* = 31, each), and healthy controls (*n* = 30). Vessel density (VD) and foveal avascular zone (FAZ) was measured with Erlangen Angio Tool.

**Results:**

Significantly lower VD were found for the DCP and ICP in Neuro-Sjögren and CIDP patients compared to healthy controls (*p* = 0.0002 and <0.0001). When group comparison was age-adjusted, these differences were not found anymore. Different frequencies of “low” retinal blood flow in each layer comparing Neuro-Sjögren and CIDP patients were not found. FAZ revealed no significant differences between patients with Neuro-Sjögren, CIDP and healthy controls.

**Conclusion:**

This study found no significant differences in VD or the foveal avascular zone between Neuro-Sjögren and CIDP patients using OCTA, suggesting that inflammatory vascular changes in the retina are uncommon in Neuro-Sjögren patients.

## Introduction

Sjögren’s syndrome is an inflammatory autoimmune disorder primarily causing inflammatory destruction of lachrymal, parotid and salivary glands.^1^ Nevertheless, systemic manifestations are also frequent, leading to arthralgia, fever, cytopenia, interstitial lung disease and glomerular nephritis among others.^1^ Neurological involvement of Sjögren’s syndrome mostly includes neuropathy (forming the entity of Neuro-Sjögren), while vasculitis, myositis and central nervous system involvement can also occur.^2^ In about 10% of cases, there is involvement of vasculitis of the small and medium blood vessels typically presenting as a rash or peripheral neuropathy.^3^ Although the etiology is not completely understood, Sjögren’s syndrome associated manifestations often respond well to immunosuppressive treatment, if applied appropriately and early in the disease course.^4–7^

Chronic inflammatory demyelinating polyneuropathy (CIDP) is also an autoimmune disorder leading to sensory deficits and motor impairment.^8^ Previous publications showed that CIDP and Sjögren’s syndrome-associated neuropathy can present with a similar phenotype, while the latter involves a significantly larger fraction of female patients and with cranial nerve impairment.^9–12^ Therefore, features that facilitate the clinical differentiation of both entities are urgently needed to prompt adequate disease management as this may differ between CIDP and Sjögren’s syndrome-associated neuropathy. Cases of vasculitis are not known for CIDP patients, which could be used as a differentiating factor, if detectable.

Optical Coherence Tomography Angiography of the eye (OCTA) is a relatively new non-invasive diagnostic tool, which visualizes the movement of tissue(s) in the neuroretina as motion-contrast images.^13–15^ In an otherwise steady environment, the blood flow in the retinal blood vessels is detected and can be shown at different depth levels.^13–15^ At different depth levels, the retinal blood circulation supply consists of different plexus (from inner retina to outer retina and retinal pigment epithelium): the superficial vascular plexus (SVP) in the retinal ganglion cell layer, the intermediate capillary plexus (ICP) above the inner nuclear layer (INL) and the deep capillary plexus under the INL (DCP).^16^ If blood vessels are affected by vasculitis (e.g. as part of a collagenous disease, such as Neuro-Sjögren) in these plexus, this can be detected by OCTA due to changes in hemodynamics typically characterized by a lower blood flow due to swelling or scarring.^17,18^ In patients suffering from Behcet’s disease or systemic lupus erythematosus, changes in the retinal microvasculature were reported for all three plexus.^19,20^ Sjögren’s syndrome and CIDP both involve damaged nerve fibers, but only Sjögren’s syndrome was previously associated with additional vascular damage mechanisms such as autoimmune vasculitis and increased cardiovascular risk.^3,12,21^ We therefore suspected that there may also be changes to the neuroretinal structures and vasculature of the macula in patients with additional Sjögren’s syndrome.

The present study aimed to evaluate changes in the small retinal blood vessels and possibly establish a new, noninvasive diagnostic tool for the clinical differentiation of patients with Sjögren’s syndrome associated neuropathy and CIDP.

## Methods

### Study design

In the present monocentric prospective study, ophthalmological examination by OCTA was performed in three groups: patients with Sjögren’s syndrome and affection of the peripheral nervous system (Neuro-Sjögren), patients with CIDP and a control group of healthy volunteers. Due to the pilot-study character of the present study, as much patients as possible were included, with the aim of including 20–30 patients with Neuro-Sjögren and CIDP similar to other studies investigating patients with collagenous diseases.^19,20^ The study has been reported in accordance with the STROBE guidelines.^22^ Data were analyzed for group differences (Neuro-Sjögren vs. CIDP vs. healthy controls) and differences of the investigated retinal layers in OCTA (DCP, ICP, SVP). Patients were recruited between April 2019 and March 2023 at the Hannover Medical School (departments of neurology and ophthalmology) from both outpatient clinics and wards. Written informed consent to study participation and data analysis was obligatory for inclusion. Exclusion criteria were advanced neurological invalidity that would prohibit participants from complementing the laborious testing. Additionally, patients with topical ophthalmic treatment other than tear substitutes were not included.

### Participants

For patients of the Neuro-Sjögren group, the diagnosis of Sjögren’s syndrome with affection of the peripheral nervous system (Neuro-Sjögren) was based on the current classification criteria.^23^ The diagnosis of Sjögren’s syndrome was prompted, when the Focus score of the following items resulted in values ⩾ 4: objective xerophthalmia (1 point) and xerostomia (1 point) on formal testing via Schirmer’s test and Saxon test, respectively; Anti-SSA/Ro-antibody-positivity (3 points) and sialadenitis with ⩾ one evident lymphocytic focus/mm^2^ (3 points).^23^ In patients with Neuro-Sjögren, ESSDAI (EULAR Sjögren’s Syndrome Disease Activity Index) and ESSPRI (EULAR Sjögren’s Syndrome Patient Report Index) were assessed to objectively evaluate disease severity and subjectively evaluate the complaints associated with dryness, fatigue, and pain.^1^ Patients of the CIDP group fulfilled the current diagnostic criteria of the European Federation of Neurological Societies/Peripheral Nerve Society (EFNS/PNS).^8^ Sjögren’s syndrome was ruled out in patients of the CIDP subgroup.

As control group, healthy persons without clinical evidence for Neuro-Sjögren or CIDP were included.

### Optical coherence tomography angiography and OCTA Erlangen-Angio tool

**Figure 1.**
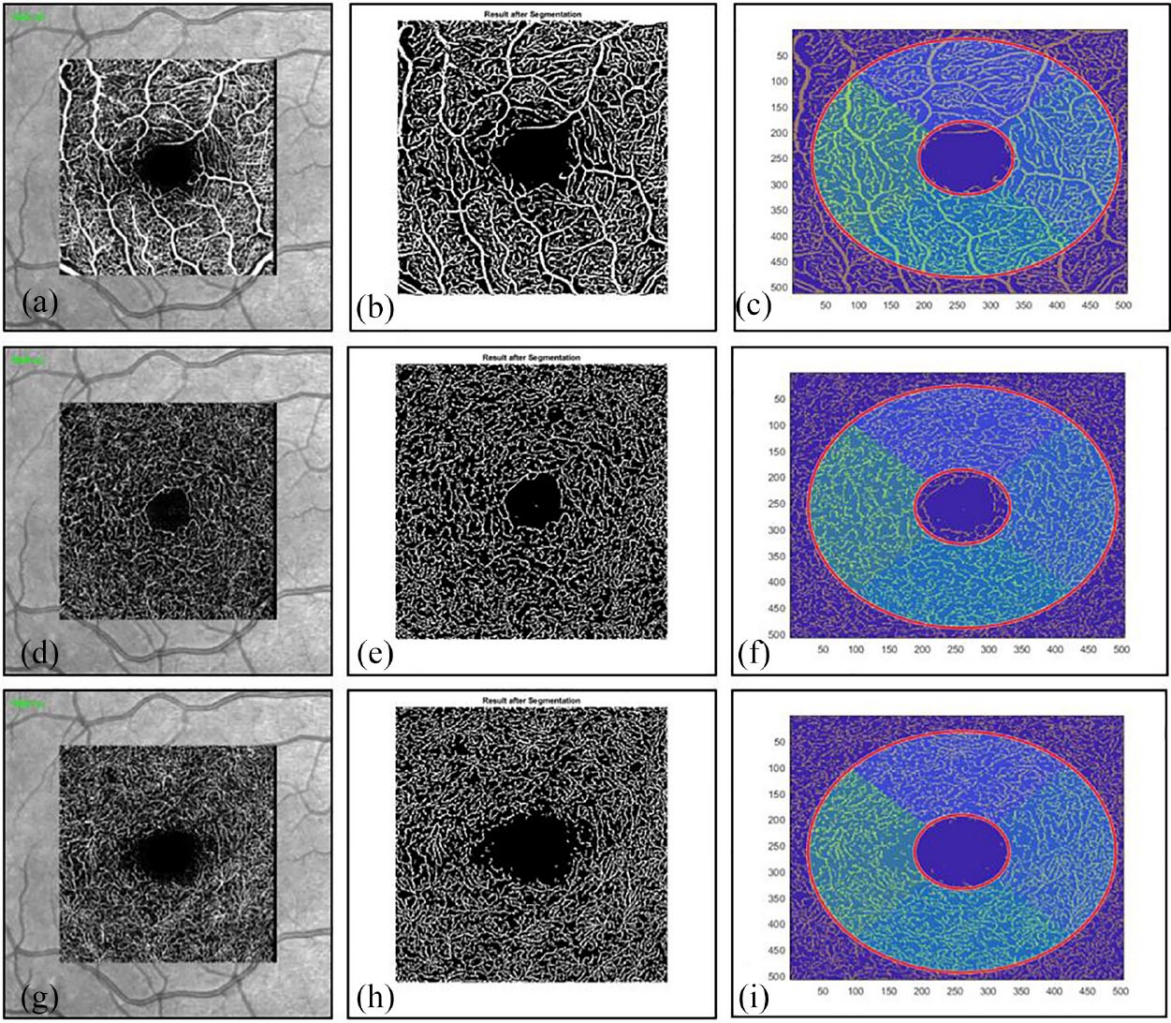
Exemplary OCTA analysis of the right eye in one patient with Neuro-Sjögren. (a)–(c) SVP analysis is shown, (d)–(f) the ICP and (g)–(i) the DCP. DCP, deep capillary plexus; ICP, intermediate capillary plexus; SVP, superficial vascular plexus.

OCTA was performed separately for both eyes of every study participant at the Heidelberg Engineering OCT SPECTRALIS (Spec-CAM-06918-S2610, Software Version 6.12.4.0) by an experienced examiner. The used OCT scan pattern consisted of 512 B-Scans and a pattern size of 2.9 mm × 2.9 mm. The automated segmentation of the different layers was performed as described by the manufacturer, whereby the OCTA scans were manually reviewed for the correct segmentation of retinal layers.^24^ Subsequently, the transverse OCTA flow images were extracted separately for the superficial (SVP), intermediate (ICP) and deep layer (DCP) of the retinal vascular plexus. Although some studies only assessed for two different plexus (SVP and DCP) and integrated the ICP into the deep layer, the reports of changes of all three plexus in patients suffering from vasculitis and collagenous diseases encouraged the analysis of the SVP, DCP and ICP as separate layers.^19,20^ In addition, different studies underlined the importance of analyzing the ICP as a separate layer, since disease-associated changes might be observed at very early stages and more pronounced as compared with the SVP and DCP.^25–27^ As possible explanation for early changes in the ICP and subsequent changes in the other layers, it was suggested that the ICP could function as an adaptative blood supply for the SVP and DCP.^25–27^ Image analysis was achieved for each eye and layer by the OCTA Erlangen-Angio Tool Version 1.0 (© University Hospital Erlangen, Department of Ophthalmology). OCTA images were manually centered, and the primary focus of the application was manually set at the center of the avascular zone as suggested by the user instructions of the OCTA Erlangen-Angio tool. Segmentation was conducted for two circles (first circle radius 0.4 mm, increment radius 0.9 mm, pixel size 0.00561 mm) divided into 90° segments and a single central segment, resulting in five segments in total. A visualization of the image analysis is shown in Figure 1. The OCTA Erlangen-Angio tool then calculated the percentage of the blood vessels (“white area”) in the retina (“total area”) of the region of interest, called vessel density (VD) as previously published.^28,29^

Further statistical analysis was performed for the mean value of the VD percentages of all 90° segments separately for both eyes.

The first image of each row shows the motion-contrast images (transverse OCTA flow images) conducted by Heidelberg Engineering OCT SPECTRALIS. The white lines represent the area in which motion was detected ergo the blood vessels. The second image of each row shows the result of the blood vessel segmentation by the OCTA Erlangen-Angio tool, where the artifacts of the first image were eliminated. There are only black and white pixels left. The third image of each row shows the segmentation into two circles and 90° segments. In the end, the mean value of the VD percentages inside the whole area of the second circle (including the inner circle) was used for statistical analysis.

### Measurement of foveal avascular zone

**Figure 2.**
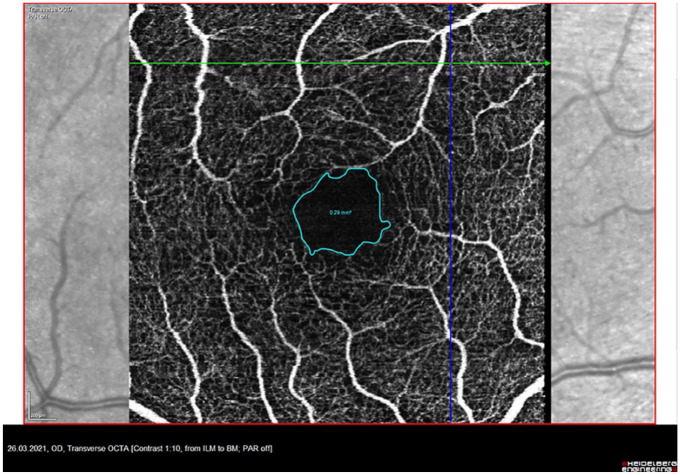
Exemplary measurement of the FAZ of the right eye in one patient with Neuro-Sjögren. FAZ, foveal avascular zone.

The same OCTA images as described above were used for measurement of the foveal avascular zone (FAZ). The transverse OCTA flow images were displayed in Heidelberg Engineering OCT SPECTRALIS (Spec-CAM-06918-S2610, Software Version 6.12.4.0). Correct segmentation of the retinal layers was confirmed. The default slabs were set to “Ret” (Retina), which includes the SVP, ICP, and DCP. Using the “draw region” tool, the avascular central region was measured manually as shown in Figure 2. To reduce examiner error and variability, only one person performed those measurements. The data are shown in mm².

The blue circle in the middle marks the foveal avascular zone. All retinal plexus are visible. This exemplary patient is the same as depicted in Figure 1.

### Statistical analysis

Shapiro–Wilk test was used to assess for parametrical distribution of decimal variables. Parametrical data were described as mean, whereas nonparametrical data were described as median, each with its range from lowest to the highest value (min–max). Group comparison was achieved via the Wilcoxon Rank sum Test for decimal data and via Chi2 test for binary data. For analysis of paired values, either the paired *t*-test (parametrically distributed values) or the Wilcoxon matched pairs test (nonparametrically distributed values) were used. For comparison of more than two groups, the post hoc test for multiple comparison error (Bonferroni correction) was performed. Statistical analysis was performed by SPSS 28.0 (IBM Co., Armonk, NY, USA). Figures were created by GraphPad Prism (version 5.02; La Jolla, CA, USA).

## Results

### Patients

A total of 31/62 patients were diagnosed with Sjögren’s syndrome according to the latest classification criteria and suffered from evident affection of the peripheral nervous system (Neuro-Sjögren). Further 31/62 patients were diagnosed with CIDP in accordance with the latest diagnostic criteria, whereas concomitant Sjögren’s syndrome was excluded in these patients. In addition, a total of 30 healthy controls were included. The controls were significantly younger than the Neuro-Sjögren and the CIDP group and the percentage of included females was significantly higher in patients with Neuro-Sjögren and healthy controls compared with the CIDP group. Neurological affection of the Neuro-Sjögren group manifested in the following manner: 9/31 (29%) suffered from cranial nerve impairment, 21/31 (68%) from motor impairment, 24/31 (77%) from sensory deficits (thereof 42% reported pain, 100% paresthesia and 58% sensory ataxia), and 4/31 (13%) from small fiber neuropathy. Further information on disease duration and investigated classification criteria for Sjögren’s syndrome is shown in Table 1.

**Table 1.** Demographical and clinical data of the investigated subgroups: Neuro-Sjögren, CIDP, and healthy controls.

	Neuro-Sjögren (*n* = 31)	CIDP (*n* = 31)	Controls (*n* = 30)	*p*-Value Neuro-Sjögren versus CIDP	*p*-Value Neuro-Sjögren versus controls	*p*-Value CIDP versus controls
Age at evaluation [years], median (min–max)	65 (40–78)	67 (24–81)	56 (20–77)	0.1464	0.0001	0.0001
Females, *n* (%)	19 (61%)	6 (19%)	18 (60%)	0.0016	0.9999	0.0016
Disease duration [months], median (min–max)	31 (1–153)	20 (1–297)	NA	0.5733	NA	NA
Cardiovascular risk factor diabetes mellitus, *n* (%)	3 (10%)	5 (16%)	NA	0.7072	NA	NA
Cardiovascular risk factor arterial hypertension, *n* (%)	14 (45%)	10 (32%)	NA	0.4345	NA	NA
*ACR / EULAR classification criteria*						
Objective xerophthalmia, *n* (%)	24 (77%)	7 (23%)	NA	<0.0001	NA	NA
Objective xerostomia, *n* (%)	12 (39%)	8 (35%)	NA	0.5653	NA	NA
SSA (Ro) antibody positivity, *n* (%)	15 (48%)	0	NA	<0.0001	NA	NA
Sialadenitis grade 3/4 by Chisholm and Mason, *n* (%)	24 (77%)	0	NA	<0.0001	NA	NA
Focus Score, median (min–max)	4 (4–8)	0 (0–2)	NA	<0.0001	NA	NA
ESSDAI total score, median (min–max)	19 (9–29)	NA	NA	NA	NA	NA
ESSPRI total score, median (min–max)	7 (3–20)	NA	NA	NA	NA	NA

ACR, American College of Rheumatology; CIDP, chronic inflammatory demyelinating polyneuropathy; ESSDAI, EULAR Sjögren’s syndrome disease activity index; ESSPRI, EULAR Sjögren’s Syndrome Patient Reported Index; EULAR, European League Against Rheumatism.

### OCTA—comparison of VD percentages and FAZ in Neuro-Sjögren, CIDP and healthy controls

**Figure 3.**
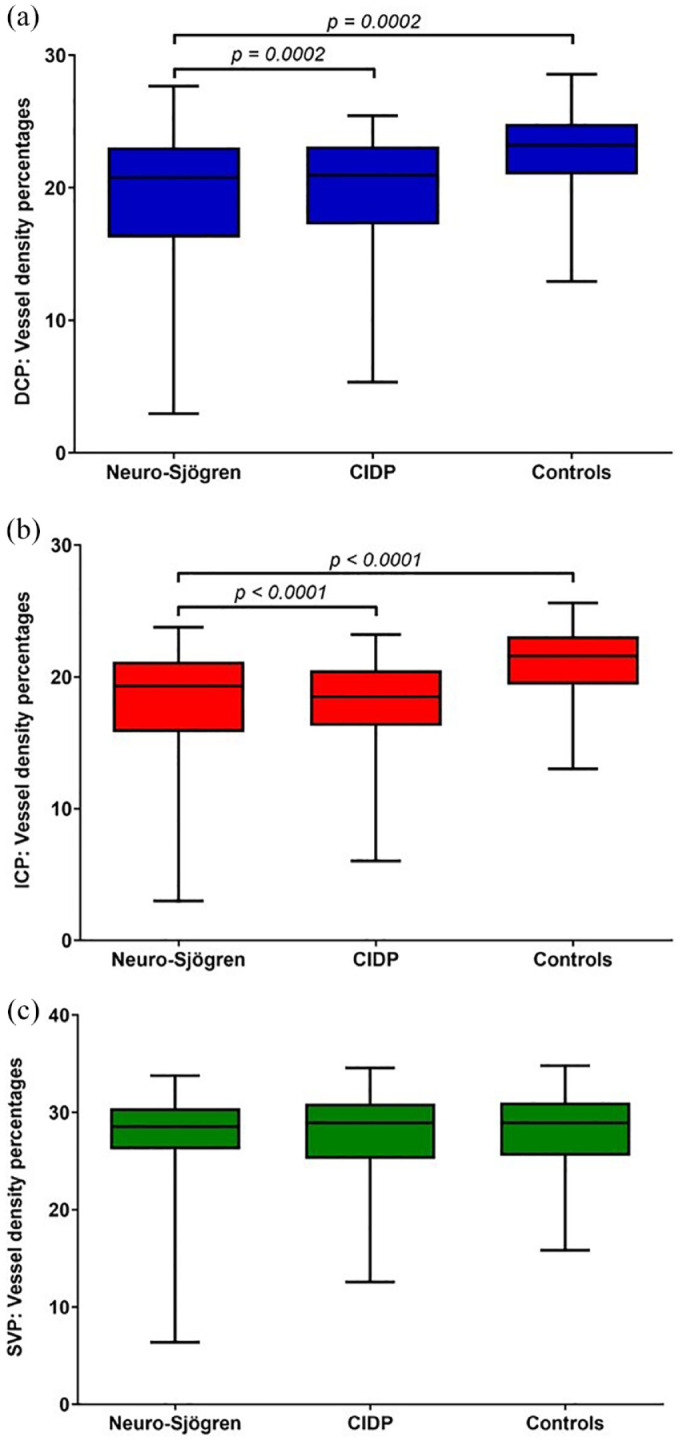
Comparison of VD percentages of both eyes of different retinal layers in patients with Neuro-Sjögren, CIDP and controls. The pooled VD percentages of different retinal layers (a) DCP, (b) ICP, (c) SVP are depicted. The comparison of different VD percentages shows significant differences in DCP and ICP layer of Neuro-Sjögren and CIDP patients compared with healthy controls. Exclusion of older patients (>70 years of age) from the Neuro-Sjögren and CIDP group resulted in nonstatistically significant differences. The level of statistical significance is shown above the line. CIDP, chronical inflammatory demyelinating polyneuropathy; DCP, deep capillary plexus; ICP, intermediate capillary plexus; SVP, superficial vascular plexus; VD, vessel density.

Comparison of pooled VD percentages of both eyes between patients with Neuro-Sjögren, CIDP, and healthy controls revealed significantly lower VD percentages in Neuro-Sjögren and CIDP patients for the DCP and ICP layer, whereas no significantly different results were found for the SVP layer. There were no statistically significant differences between Neuro-Sjögren and CIDP patients in any layer (DCP: *p* = 0.8943; ICP: *p* = 0.3159; SVP: *p* = 0.7439) as shown in Figure 3. Re-analyzing the VD percentages with exclusion of all patients with Neuro-Sjögren and CIDP that are older than 70 years of age (Neuro-Sjögren *n* = 23, CIDP *n* = 19), comparison with healthy controls resulted in no statistically significant differences for DCP (*p* = 0.1753) and ICP (*p* = 0.0532).

Concerning FAZ, pooled data of both eyes were compared between patients with Neuro-Sjögren, CIDP and healthy controls. Here, no significant differences were found (Neuro-Sjögren: median 0.245 mm^2^, CIDP: median 0.260 mm^2^, controls: median 0.260 mm^2^, *p* = 0.8494).

### No detection of Neuro-Sjögren-specific vasculitis by OCTA

Since up to 10% of the patients with Sjögren’s syndrome are expected to present with vasculitic changes of the small and medium blood vessels (as indicated by changes in hemodynamics with a lower blood flow due to swelling or scarring), it was investigated, whether the Neuro-Sjögren patients with the lowest retinal blood flow revealed significantly lower VD percentages compared to the remaining patients with Neuro-Sjögren as possible signs for Neuro-Sjögren-associated vasculitis. For the DCP, Neuro-Sjögren patients with a VD percentage below 15% (*n* = 6) revealed significantly lower VD percentages than the rest of the Neuro-Sjögren patients (*p* = 0.0003), the CIDP control group (*p* = 0.0007) and the healthy controls (*p* = 0.0004). For the ICP, Neuro-Sjögren patients with a VD percentage below 14% (*n* = 5) revealed significantly lower VD percentages than the rest of the Neuro-Sjögren patients (*p* = 0.0008), the CIDP control group (*p* = 0.0078), and the healthy controls (*p* = 0.001). For the SVP, Neuro-Sjögren patients with a VD percentage below 24% (*n* = 6) revealed significantly lower VD percentages than the rest of the Neuro-Sjögren patients (*p* = 0.0007), the CIDP control group (*p* = 0.0024), and the healthy controls (*p* = 0.0009).

Comparison of the VD of these Neuro-Sjögren patients with the lowest retinal blood flow with CIDP patients with VD below the same cut-offs revealed no significant differences neither for the proportion of patients with low VD for each layer (CIDP DCP < 15%: *n* = 6; CIDP ICP < 14%: *n* = 7; CIDP SVP < 24%: *n* = 8), nor for VD percentages themselves (DCP: *p* = 0.1785, ICP: *p* = 0.9547; SVP: *p* = 0.1405).

### OCTA—correlations with disease activity in patients with Neuro-Sjögren

Comparison of VD percentages of the retinal layers of both eyes combined revealed significantly lower DCP and ICP values in patients with Neuro-Sjögren with high disease activity (ESSDAI > 20; *n* = 7) versus healthy controls (DCP (mean 16.5%, *p* = 0.0070) and ICP (mean 15%, *p* = 0.0004)). However, correlations of VD percentages of the retinal layers of both eyes combined with measures of disease activity did not reveal statistical significance. Linear regressions of VD percentages of the DCP, ICP, and SVP with ESSDAI revealed p-values between 0.2913 and 0.8967 (*p*-values for correlation between 0.1732 and 0.4444) and with ESSPRI *p*-values between 0.1336 and 0.3491 (*p*-values for correlation between 0.1167 and 0.2269).

Comparison of ESSDAI and ESSPRI scores of Neuro-Sjögren patients with high and low VD percentages of the retinal layers (DCP cut-off 15%, ICP cut-off 14%, SVP cut-off 24%) did not reveal statistical significances (*p*-values between 0.3493 and 0.6679). SSA antibody positivity in Neuro-Sjögren patients with high or low disease activity regarding ESSDAI score did not reveal a significant difference.

The same statistical analyses were also employed for FAZ, but no statistical significant results found (data not shown).

## Discussion

Sjögren’s syndrome-associated neuropathy (Neuro-Sjögren) and CIDP constitute different disease entities, which are clinically difficult to differentiate but still partly differ in disease management strategies. Since Sjögren’s syndrome is partly associated with vasculitis of the small and medium blood vessels, we assessed if visualization of the retinal vasculature by OCTA might facilitate the clinical differentiation of both entities.

*In the present study, significant difference in VD percentages between Neuro-Sjögren, CIDP and healthy controls were observed for the DCP and ICP layer but not the SVP (p* *=* *0.0002 and <0.0001, respectively).* In cases of vasculitis, the blood flow would be expected to be lower due to swelling or scarring and subsequently the VD to be reduced.^17,18^ Therefore, it might be speculated that this finding is a sign of retinal vasculitis, since ocular affection of systemic vasculitis is described in patients with Sjögren’s syndrome.^3,30,31^ Nevertheless, technical and/or methodological deficits of OCTA imaging have also to be considered. In other publications of OCTA imaging in patients with known retinal vasculitis, the vascular changes of those patients were not sufficiently identifiable in OCTA in comparison to “standard of care” fluorescein angiography. Therefore, OCTA has not replaced fluorescein angiography as a diagnostic tool for retinal vasculitis.^32^ Another explanation might be that patients with Sjögren’s syndrome and evident SSA (Ro) and SSB (La) antibodies have been reported to more frequently present with vasculitis while our studied Neuro-Sjögren cohort consisted of 48% seropositive patients only.^33^ However, vasculitis in patients with Sjögren’s syndrome is a rather scarce finding, thus only a small number of the included patients would be statistically expected to suffer from this manifestation.^3,34–36^ In addition, neither the typical presentation with a skin rash affecting the smaller blood vessels, nor any evidence of previous episodes of vasculitis possibly leading to scarring and reduced blood flow was observed in any of the included Neuro-Sjögren patients.^3^ However, the differences observed in the Neuro-Sjögren and CIDP group might be rather age-associated and/or unspecific, since no differences between Neuro-Sjögren and CIDP patients was observed, the differences were limited to the DCP and ICP only (but not the SVP), and comparison of the VD percentages of the DCP and ICP with healthy controls was not statistically significant, when patients older than 70 years of age were excluded.

Similarly, a small proportion of Neuro-Sjögren patients with significantly lower flow density percentages compared with healthy controls was identified (*n* = 6 for DCP, *n* = 5 for ICP and SVP) possibly associated with vasculitis in these patients. However, applying the same relative cut-offs for a “low” flow density in patients with CIDP, no significant differences for the proportion of patients or for VD percentages compared to Neuro-Sjögren patients could have been observed. Therefore, it was concluded that the low flow density percentages in these patients do not reflect vasculitic changes, but rather the range of “normal” results. In line with this conclusion, no differences in ESSPRI and ESSDAI scores of Neuro-Sjögren patients with “high” and “low” flow density percentages could have been identified and no significant correlation of flow density percentages with ESSDAI and ESSPRI scores were found.

Recently, an increased cardiovascular risk in patients with Sjögren’s syndrome was reported.^21^ The findings of the present study cannot support this hypothesis, which might be due to varied reasons. On the one hand, the small amount of investigated Neuro-Sjögren patients, which is partly caused by the low prevalence of the disorder itself, might be explanatory.^1,23^ On the other hand, the different clinical phenotypes investigated might explain the observed discrepancy. An increased cardiovascular risk was mostly reported in patients with Sjögren’s syndrome without neurological involvement, whereas the present study investigated only patients with peripheral neurological involvement (Neuro-Sjögren).^21^ Since Neuro-Sjögren apparently affects demographically different patient groups than Sjögren’s syndrome without neurological involvement, it might be speculated that Neuro-Sjögren patients represent a clinically different phenotype of the disease. Besides the manifestation in more male and older patients, this phenotype might also be characterized by a lower cardiovascular risk compared with Sjögren’s syndrome patients without neurological involvement.^21^ Lastly, alterations of FAZ were shown to be associated with arterial hypertension and diabetes mellitus in different studies.^37–39^ In the present study, despite differences in age between the Neuro-Sjögren group, the CIDP group, and the healthy controls group, no significant differences in FAZ were found. This finding might also point toward an equal cardiovascular risk in all investigated groups in this study.

However, this study is not free of limitations, such as the limited number of included patients. Although the number of included patients in this pilot-study is low, it was clearly found that OCTA did not differentiate between Neuro-Sjögren and CIDP. Another limitation of the study is the usage of OCTA itself, since it is not free of errors and artifacts. To minimize the influence of potential artifacts using OCTA, results of both eyes of all patients were pooled. Lastly, the statistically significant differences in sex and age of the healthy controls group harbor the risk of a selection bias as possible explanation for the found differences between Neuro-Sjögren and CIDP patients and healthy controls.

In conclusion, our data suggest no specific or disease-related changes in the VD in patients with Neuro-Sjögren or CIDP. While it is a noninvasive test, differentiation between Sjögren-associated neuropathy and CIDP was not facilitated by OCTA measurements in our cohort. Further studies with larger cohorts may be necessary to investigate specific vascular changes in Neuro-Sjögren patients, who have suffered of vasculitis of small and medium blood vessels.

## Supplemental Material

sj-docx-1-oed-10.1177_25158414241294024 – Supplemental material for Optical coherence tomography angiography to assess for retinal vascular changes in Neuro-Sjögren Supplemental material, sj-docx-1-oed-10.1177_25158414241294024 for Optical coherence tomography angiography to assess for retinal vascular changes in Neuro-Sjögren by Melanie Haar, Franz Felix Konen, Marten A. Gehlhaar, Irene Oluwatoba-Popoola, Emilia Donicova, Marija Wachsmann, Ahmed Lubbad, Katerina Hufendiek, Amelie Pielen, Bettina Hohberger, Christian Mardin, Stefan Gingele, Nils K. Prenzler, Diana Ernst, Torsten Witte, Carsten Framme, Thomas Skripuletz, Tabea Seeliger and Anna Bajor in Therapeutic Advances in Ophthalmology

## Declarations

**Ethics approval and consent to participate:** The study was conducted in accordance with the Declaration of Helsinki and approved by the Local Ethics Committee of the Hannover Medical School (No. 8297_BO_S_2019; 25. February 2019; No. 7687; 12 March 2018). Written informed consent was obtained from all subjects involved in the study.

**Consent for publication:** Written informed consent was obtained from all subjects involved in the study.

**Competing interests:** The authors have no relevant financial or nonfinancial interests to disclose. No external funding was involved in the present study. Outside the submitted work, the authors received honoraria for lectures, travel grants, or research grants: M.H. participated in an Advisory Board of Roche, received travel grants from Janssen Cilag, Genentech, Iveric Bio (now Astellas Pharma), and Bayer and honoraria for a lecture at an ophthalmologist’s office. F.F.K. received travel grants from Merck and Novartis. T.S. received financial support for congress fees by Abbvie. A.P. participated in Advisory Boards of Bayer, Novartis, and Roche and received honoraria for lectures and presentations by Novartis, Bayer, and Roche as well as consulting fees from the Roche National Study Committee. S.G. received research support from Alnylam Pharmaceuticals, CSL Behring, Else Kröner Fresenius Foundation, Deutsche Forschungsgemeinschaft, and Hannover Biomedical Research School (HBRS) and consulting and/or speaker honoraria or travel expenses from consulting and/or speaker honoraria from Alexion, Alnylam Pharmaceuticals, AstraZeneca, CSL Behring, GSK, Pfizer and Merck all outside the submitted work. D.E. received presentation honoraria from Abbvie, Amgen, BMS, Chugai, Cilag-Janssen, Galapagos, GSK, Medac, Lilly, Pfizer, Novartis, Roche and participated in advisory boards for Abbvie, Galapagos, Amgen and Novartis. T.W. received honoraria for lectures by Abbvie, BMS, Chugai, Galapagos, Janssen, Lilly, Pfizer, UCB, and Roche. Th.S. reports research support from Alnylam Pharmaceuticals, CSL Behring, Novartis; honoraria for lectures and travel expenses for attending meetings from Alexion, Alnylam Pharmaceuticals, argenx, Bayer Vital, Biogen, Bristol Myers Squibb, Celgene, Centogene, CSL Behring, Euroimmun, Grifols, Hexal AG, Horizon, Janssen-Cilag, Merck Serono, Novartis, Pfizer, Roche, Sanofi, Siemens, Swedish Orphan Biovitrum, Teva, Viatris; consultant fees from Alexion, Alnylam Pharmaceuticals, Biogen, Centogene, CSL Behring, Grifols, Hexal AG, Janssen-Cilag, Merck Serono, Novartis, Roche, Sanofi, Swedish Orphan Biovitrum, Viatris. I.O.-P. received travel grants from Genentech. B.H. received grants or contracts, support for attending meetings and/or travel and equipment, materials, drugs, medical writing, gifts or other services from Heidelberg Engineering. C.M. received consulting fees and payment or honoraria for lectures, presentations, speakers bureaus, manuscript writing or educational events from Heidelberg Engineering. Th.S. reports honoraria for lectures and travel expenses for attending meetings from Alexion, Alnylam Pharmaceuticals, argenx, Bayer Vital, Biogen, Celgene, Centogene, CSL Behring, Euroimmun, Grifols, Hexal AG, Janssen-Cilag, Merck Serono, Novartis, Pfizer, Roche, Sanofi, Siemens, Swedish Orphan Biovitrum, Teva, Viatris. His research is supported by the German Ministry for Education and Research (BMBF), Bristol-Myers Squibb Foundation for Immuno-Oncology, Claudia von Schilling Foundation for Breast Cancer Research, Else Kröner Fresenius Foundation, Hannover Biomedical Research School (HBRS), Alnylam Pharmaceuticals, CSL Behring, Novartis, Sanofi Genzyme, VHV Foundation. M.A.G. received travel grants from Iveric Bio (now Astellas Pharma) and travel grants and congress fees from Ophtec. N.P. received support for attending meetings and/or travel from Med-El Innsbruck, Austria. E.D., M.W., C.F., A.B., A.L., and K.H. have nothing to disclose.

**Availability of data and materials:** Data are available upon reasonable request from the corresponding author.
